# Identification of a 10-protein plasma signature for preclinical gout risk beyond serum urate: a robustness-first proteomic study in 33,147 normouricaemic individuals

**DOI:** 10.1186/s12967-026-08505-3

**Published:** 2026-06-24

**Authors:** Yue Yuan, Huan Zhang, Kun Zhang, Mingzhu Wang, Hao Yan, Junyang Zhang, Chengping Wen, Zheshan Guo, Lin Huang, Zhaoxiang Wang

**Affiliations:** 1 Hangzhou Red Cross Hospital, Hangzhou, 310003 China; 2 Research Center for Life Sciences Computing, Zhejiang Lab, Hangzhou, 311100 China; 3 State Key Laboratory of Digital Medical Engineering, Key Laboratory of Biomedical Engineering of Hainan Province, School of Biomedical Engineering, Hainan University, Sanya, 572024 China; 4 School of Basic Medical Sciences, Zhejiang Chinese Medical University, Hangzhou, 310053 China; 5 Key Lab of Biomedical Engineering for Ministry of Education, College of Biomedical Engineering and Instrument Science, Zhejiang University, Hangzhou, 310027 China; 6 Sanya Research Institute of Hainan University, Sanya, Hainan 572024 China; 7 Zhejiang Hospital of Integrated Traditional Chinese and Western Medicine, Hangzhou, 310003 China; 8 Hangzhou Integrative Medicine Hospital Affiliated to Zhejiang Chinese Medical University, Hangzhou, 310003 China

**Keywords:** Gout, Plasma proteomics, Serum urate, Normouricaemia, Machine learning, Preclinical biomarkers, UK Biobank

## Abstract

**Background:**

Gout risk assessment is currently reactive, relying on hyperuricaemia. However, a significant clinical gap exists for individuals who develop gout despite maintaining “normal” serum urate levels. We aimed to identify a plasma proteomic signature that defines a susceptibility state for gout years before clinical onset, specifically within individuals who are normouricaemic at baseline.

**Methods:**

We analyzed large-scale plasma proteomic data (2,920 proteins) from 33,147 normouricaemic individuals (baseline serum urate < 360 μmol/L) in the UK Biobank. To ensure findings were not artifacts of specific algorithms, we employed a “robustness-first” machine-learning framework encompassing 756 model configurations. Longitudinal protein trajectories were traced up to 15 years prior to diagnosis. Incident osteoarthritis was used as a negative control to evaluate specificity.

**Results:**

A 10-protein signature (including VSIG4, UBQLN3, and MB) predicted incident gout with high discrimination (ROC-AUC = 0.892). This signature identified a high-risk subgroup with a 4.46-fold increased hazard, independent of baseline serum urate and conventional risk factors. Notably, protein trajectories diverged between future cases and controls 10–15 years before clinical diagnosis, even while serum urate remained within the normal range. The signature showed minimal discrimination for osteoarthritis (AUC ≈ 0.60).

**Conclusions:**

In 33,147 normouricaemic individuals, plasma proteomics identifies a stable preclinical susceptibility state for gout that exists beyond the traditional serum urate paradigm. This 10-protein signature enables early risk stratification and offers a significant window for precision prevention a decade before clinical onset.

**Supplementary information:**

The online version contains supplementary material available at https://doi.org/10.1186/s12967-026-08505-3.

## Introduction

Gout is the most prevalent inflammatory arthritis, imposing a rapidly increasing clinical and socioeconomic burden worldwide [1, 2]. While historically viewed as a simple consequence of monosodium urate (MSU) crystal deposition, gout is now recognized as a complex systemic disorder driven by intricate genetic, metabolic, and immune networks [3–5]. However, current risk assessment strategies remain largely reactive, relying on the detection of established hyperuricaemia or the onset of clinical symptoms [6, 7]. These events often mark the culmination rather than the beginning of disease pathology, missing the optimal window for preventive intervention before irreversible tissue injury occurs [8].

A significant clinical paradox persists: hyperuricaemia is necessary but insufficient for gout, and a substantial proportion of individuals develop the disease despite maintaining “normal” serum urate levels [9–12]. This suggests that a prolonged preclinical phase, characterized by subtle molecular perturbations, precedes overt biochemical aberrations [13–16]. While high-throughput proteomics has identified early circulating signatures for various chronic diseases, the molecular landscape specifically preceding gout onset, particularly in individuals without established hyperuricaemia, remains poorly characterized [17–22]. Most epidemiologic studies have focused on hyperuricaemic cohorts, obscuring the early pathogenic mechanisms that operate independently of, or prior to, urate saturation [1, 4, 23].

To address this gap, we hypothesized that distinct plasma proteomic profiles can define a “pre-hyperuricaemia” risk state and predict future gout even among individuals with normal baseline serum urate. Leveraging large-scale data from 33,147 participants in the UK Biobank, we applied a robustness-first machine-learning framework, evaluating 756 model configurations, to identify a parsimonious proteomic signature of incident gout. By integrating multi-stage feature selection with long-term time-to-event analyses, we sought to characterize the temporal evolution of gout risk up to 15 years prior to diagnosis. Complementary negative control analyses for osteoarthritis were performed to ensure outcome specificity. This study aims to position plasma proteomics as a window into the preclinical biology of gout, offering a framework for early risk stratification beyond the traditional serum urate paradigm.

## Methods

### Study population and outcome definition

This study was conducted using the UK Biobank (UKB) cohort, a large prospective study with extensive proteomic, clinical, and longitudinal follow-up data [24]. UK Biobank was conducted in accordance with the Declaration of Helsinki and approved by the North West Multi-centre Research Ethics Committee (Ref. 11/NW/0382). All participants provided written informed consent. The current analyses were conducted under UKB application number 584,010.

**Fig. 1:**
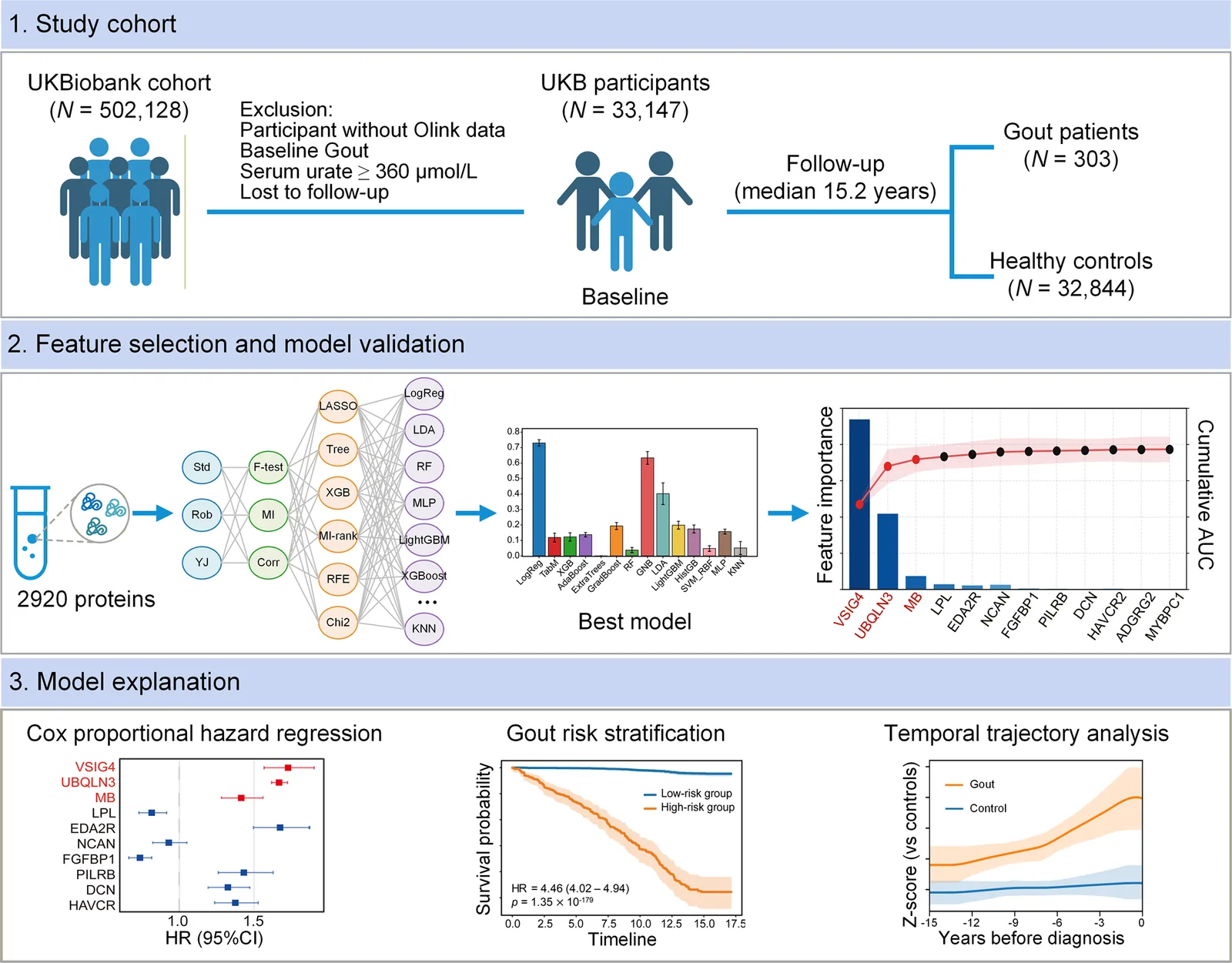
Overview of the study design, modeling pipeline, and downstream analyses. UK Biobank participants were included after exclusion of individuals without Olink data, prevalent gout, baseline serum urate ≥ 360 μmol/L, or loss to follow-up, yielding 33,147 participants at baseline. Over a median follow-up of 15.2 years, 303 incident gout cases and 32,844 controls were identified. A total of 2,920 plasma proteins were analyzed using multiple feature-selection strategies and machine-learning models to identify the optimal predictor. The selected proteomic signature was further evaluated using Cox proportional hazards regression, risk stratification, and longitudinal trajectory analysis

We included participants who had available baseline plasma proteomic measurements (Fig. 1). To investigate early biological determinants of gout risk beyond traditional hyperuricaemia, we restricted the population to normouricaemic individuals with normal serum urate levels at baseline (<360 μmol/L) and no prior history of gout [4]. By excluding those with baseline hyperuricaemia or prevalent gout, this design enabled us to assess proteomic predictors of future gout in an ostensibly low-risk cohort, reducing confounding by pre-existing metabolic derangements.

Participants were followed from baseline assessment until the first occurrence of gout (incident gout), loss to follow-up, death, or end of study follow-up (2024–05-31). The median follow-up time was 15.2 years (IQR: 14.4–15.9 years). Incident gout cases were identified through linked health records using International Classification of Diseases (ICD-10) diagnostic codes for gout, supplemented by hospital inpatient data. We constructed three outcome definitions to capture different temporal windows of gout onset: (a) overall incident gout (any time during follow-up), (b) short-term gout (onset within ≤ 5 years of baseline), and (c) long-term gout (onset > 5 years after baseline). Unless specified otherwise, the primary outcome for analysis was overall incident gout.

### Plasma proteomic profiling and quality control

Baseline blood samples were processed for high-throughput proteomic profiling. Plasma was aliquoted and stored at −80 °C until analysis. Protein quantification was performed by Olink (Uppsala, Sweden) using the Olink Explore 3072 proximity extension assay (PEA) platform [25]. This antibody-based platform measured 2,941 protein analytes corresponding to 2,923 unique proteins, spanning four panels (inflammation, cardiometabolic, neurology, and oncology). The PEA technology enables sensitive detection of low-abundance proteins from small sample volumes [25]. Standard quality controls were applied by Olink: inter-plate coefficient of variation (CV) < 20% and intra-plate CV < 10% for all assays. Protein levels were reported on a log2 scale as Normalized Protein eXpression (NPX) values [14, 26].

We implemented stringent quality control (QC) in line with the UKB proteomics pipeline [17, 18]. Samples with > 30% of proteins missing were excluded to ensure data completeness. Proteins with a high fraction of missing values or technical outliers were removed according to pre-established criteria. After QC, 2,920 proteins remained for analysis, reflecting the high call rate of the platform [14, 17]. Missing protein values (which were rare after sample QC) were imputed by a low-risk method (median imputation) within each assay panel to avoid bias. The final analytic dataset consisted of 33,147 individuals (all normouricaemic at baseline) with quantitative levels for 2,920 plasma proteins.

### Data preprocessing and feature selection

To ensure robustness of findings, we processed the proteomic data using multiple preprocessing techniques in parallel. In particular, we generated three versions of the dataset: (1) a median-centered standard scaling (Std) was applied, preserving relative variability while reducing sensitivity to extreme values in centering. (2) a median-centered robust scaling (Rob) was used, scaling features by the interquartile range (IQR) to improve robustness to outliers and heavy-tailed distributions. (3) a median-centered Yeo–Johnson (YJ) transformation was performed, yielding approximately symmetric distributions while preserving rank information [27, 28]. Each method aimed to normalize distributions and mitigate technical variation while preserving biological signal. All continuous phenotypic covariates were similarly normalized to ensure comparability, although only proteomic features were used for model building. By retaining multiple preprocessing schemes, we could later perform sensitivity analyses to confirm that our results were not an artifact of any single data transformation.

Given the high dimensionality relative to the number of gout cases, we employed a multi-step feature selection to reduce dimensionality and guard against overfitting [28, 29]. First, we applied three complementary univariate filtering methods: (a) ANOVA *F*-test; (b) Mutual Information (MI); and (c) Correlation filtering (Corr). The top ~250 proteins from each method were selected as candidates. Next, each candidate set was subjected to six multivariate techniques: LASSO regularization, tree-based importance (Tree), XGBoost importance (XGB), mutual information ranking (MI-rank), recursive feature elimination (RFE), and chi-square filtering (Chi2). In total, across three preprocessing schemes, three initial filters, and six secondary methods, we generated 54 distinct feature sets. This exhaustive consensus-based approach prioritized the stability of feature selection, identifying proteins that consistently emerged across methods as robust predictors while mitigating the biases of any single algorithm. 

### Robustness-first framework for biomarker discovery

To mitigate algorithmic bias and ensure the stability of the identified proteomic signature, we implemented a robustness-first framework. Instead of optimizing a single model, we systematically evaluated protein performance across 756 distinct model configurations, encompassing 54 feature-selection pipelines and 14 classification algorithms, to ensure that the emerging signals represented genuine biological markers rather than artifacts of a specific mathematical method.

### Machine learning model development

We implemented 14 classification algorithms spanning linear, non-linear, ensemble, and neural network methods, including Logistic Regression (LogReg), Random Forest (RF), XGBoost, LightGBM, and TabM, among others. Each model was trained using five-fold cross-validation, resulting in a comprehensive evaluation of 756 configurations to assess how performance remained stable across modeling choices.

Model performance was evaluated via repeated cross-validation. Standard classification metrics were calculated, including ROC-AUC, PR-AUC, accuracy, precision, and recall (sensitivity). Class imbalance was handled using class-weighting and algorithmic adjustments. Given the clinical aim of early detection, we prioritized sensitivity (recall) in selecting an optimal model while maintaining a high specificity threshold. We first filtered for models with mean ROC-AUC > 0.85 and then identified the configuration with the highest recall to maximize early case detection.

Starting from the optimal model’s feature set, we performed forward feature selection to derive a parsimonious predictive protein signature. Proteins were added sequentially based on their contribution to ROC-AUC, using DeLong’s test to confirm significant improvements (*p* < 0.05). To interpret the final model, we applied Shapley Additive Explanations (SHAP) to quantify each feature’s contribution to individual predictions and visualize the direction and magnitude of protein effects [30].

### Survival analysis and risk stratification

To validate the prognostic value on a continuous time-to-event scale, we performed Cox proportional hazards regression [31, 32]. Three models with increasing adjustment were fitted: Model 1 adjusted for age, sex, and race; Model 2 added BMI, education, smoking, and alcohol; Model 3 further adjusted for baseline serum urate concentration to evaluate the incremental predictive value of proteomics beyond the traditional biochemical marker.

Participants were stratified into high- and low-risk groups based on the proteomic risk score using a cutoff that maximized the Youden index. Kaplan–Meier curves were constructed to compare gout-free survival, with differences assessed by the log-rank test. Hazard ratios (HRs) for high versus low risk were estimated under both protein-only and covariate-adjusted scenarios [32, 33].

### Temporal trajectory analysis

To explore how selected protein levels change leading up to diagnosis, we conducted a nested case–control temporal trend analysis [27, 34]. Each case was matched with up to two controls based on age, sex, ethnicity, and baseline characteristics, including baseline serum urate levels. We traced the trajectories of the top-ranked proteins over 15 years using serial plasma samples and fit locally weighted scatterplot smoothing (LOESS) curves to visualize trends. This allowed us to determine when proteomic profiles began to diverge between future cases and controls prior to clinical diagnosis.

### Negative control outcome analysis

To evaluate outcome specificity and ensure the identified signature reflected a gout-specific risk state rather than nonspecific musculoskeletal inflammation, we performed a negative control analysis using incident osteoarthritis (OA). Analyses were restricted to participants with normal baseline serum urate. The same protein signature and full modeling framework were applied to incident OA to assess whether the signature generalized to unrelated musculoskeletal outcomes.

### Data availability

This study used de-identified data from UK Biobank under application 584,010. The proteomic and phenotypic datasets are not publicly available due to institutional and participant privacy constraints but can be accessed via UK Biobank by qualified researchers upon application. Analysis code can be made available upon reasonable request.

## Results

### Baseline characteristics of the study population

A total of 33,147 participants were included in the final analysis after excluding individuals with missing Olink proteomic data, prevalent gout, missing follow-up information, or baseline hyperuricaemia (Fig. 1). Consequently, all included participants were normouricaemic at baseline, enabling the prospective evaluation of gout risk independent of the traditional serum urate paradigm. During a median follow-up of 15.2 years (IQR: 14.4–15.9), 303 participants developed incident gout, while 32,844 remained gout-free (healthy controls).

Baseline characteristics are summarized in Table 1. Compared to controls, participants who subsequently developed gout were significantly older (mean age: 61.5 vs. 56.8 years, *p* < 0.001) and were predominantly male, with a substantially lower proportion of women (26.1% vs. 66.1%, *p* < 0.001). Although all participants maintained normal serum urate levels at baseline, those who developed gout exhibited higher mean concentrations (292.8 vs. 273.5 μmol/L, *p* < 0.001), and a higher mean BMI (30.2 vs. 26.8 kg/m^2^, *p* < 0.001).The majority of participants were of White ethnicity (>93% in both groups), with no significant difference observed between cases and controls (*p* = 0.786). Socioeconomic disparities were noted, with a lower proportion of college-educated individuals among incident gout cases (22.8% vs. 31.9%, *p* < 0.001). No significant differences were observed regarding smoking status (*p* = 0.393) or alcohol consumption (*p* = 0.908).

**Table 1:** Characteristic of the participants at baseline

	Overall (*N* = 33147)	Gout patients (*N* = 303)	Healthy controls (*N* = 32844)	*P* **values**
Age (mean, SD)	56.83 (8.20)	61.548 (6.71)	56.79 (8.20)	<0.001
Female	21798 (65.8%)	79（26.1%）	21719（66.1%）	<0.001
BMI (mea n, SD)	26.84 (4.59)	30.24 (5.65)	26.81 (4.57)	<0.001
Ethnic, white	30975（93.6%）	280（93.0%）	30695（93.6%）	0.786
Education level				<0.001
College degree	10437（31.9%）	69（22.8%）	10368（31.9%）	
Other degree	22331（68.1%）	233（77.2%）	22098（68.1%）	
Current smoker	3642（11.0%）	28（9.3%）	3614（11.0%）	0.393
Current drinker	29987（90.6%）	272（90.4%）	29715（90.6%）	0.908
Serum Urate	273.60 (52.04)	292.79(45.71)	273.46 (52.07)	<0.001

*p* values were estimated by student *t* test for continuous variables and χ^2^ test for discrete variables

### Proteomic feature selection and model screening

**Fig. 2:**
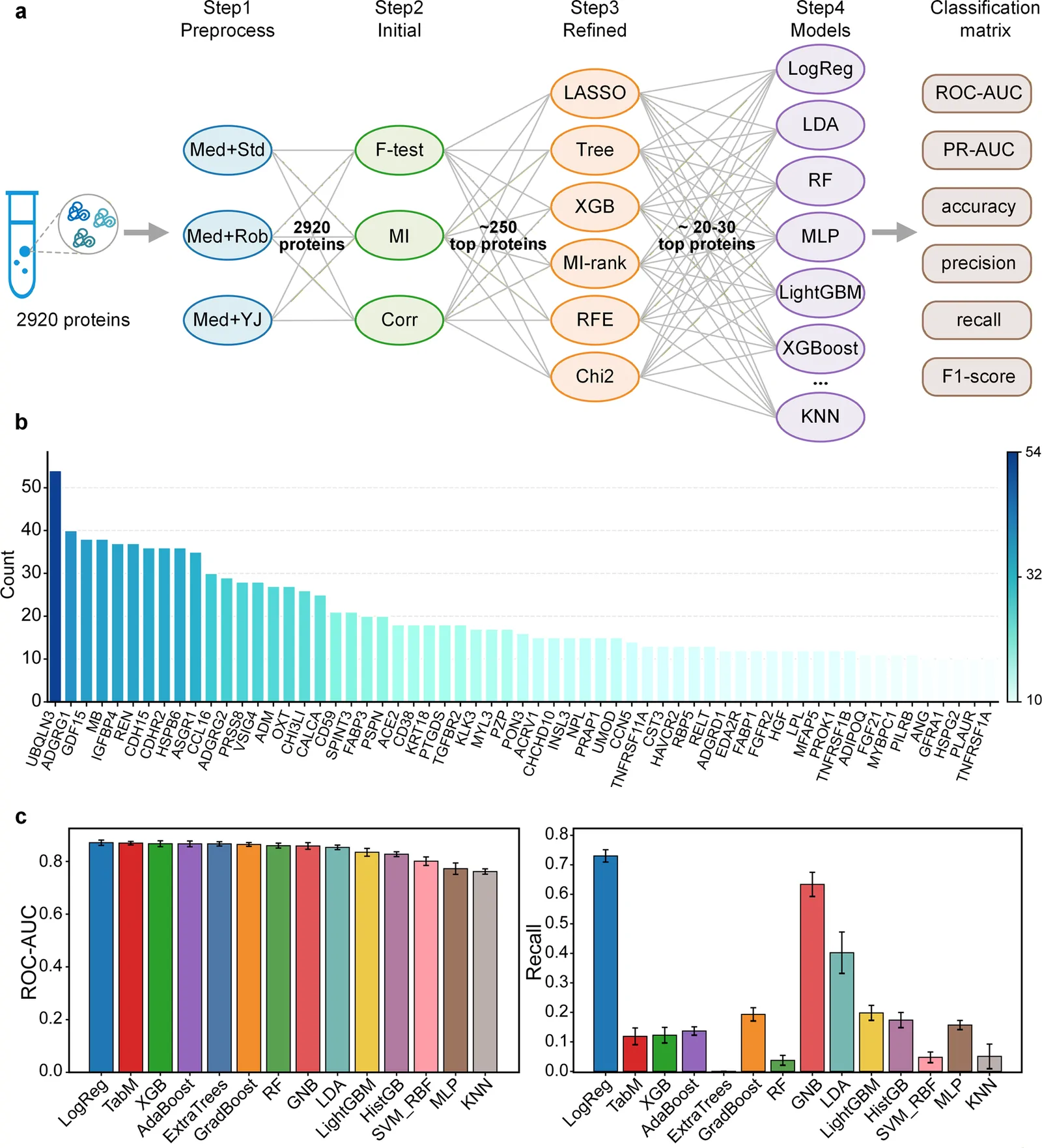
Feature selection and model evaluation. **a**, Schematic overview of the multi-stage modeling pipeline. A total of 2,920 plasma proteins were processed using multiple preprocessing strategies, followed by initial and refined feature selection steps, and subsequently evaluated across a panel of machine-learning classifiers. Model performance was assessed using multiple classification metrics. **b**, Frequency of protein selection across different feature-selection strategies, showing the number of times each protein was retained. **c**, Predictive performance of different classifiers, evaluated by cross-validated ROC-AUC and recall

To identify highly robust predictors of gout risk, we implemented a systematic machine-learning pipeline (Fig. 2a). Three preprocessing strategies were applied to the raw expression matrix, followed by univariate filtering, reducing the feature space to approximately 250 candidate proteins per dataset. These candidates were further refined using six complementary feature-selection methods, yielding optimized subsets of 20–30 proteins.

Across all 54 feature-selection configurations, a total of 61 proteins were selected in at least 10 independent selections (Fig. 2b). Notably, UBQLN3 was retained across all 54 schemes, followed by high selection frequencies for ADGRG1, GDF15, MB, and IGFBP4 (Supplementary Table 1).

Fourteen machine-learning classifiers were then trained independently on each optimized feature subset. In total, the combination of three preprocessing strategies, three initial filters, six refined feature-selection methods, and fourteen classifiers resulted in 756 distinct model configurations, enabling a systematic assessment of model robustness across preprocessing, feature-selection, and algorithmic choices (Fig. 2a).

Five-fold cross-validation demonstrated robust predictive performance across the evaluated models, with 451 of the 758 configurations achieving a mean ROC-AUC greater than 0.85. Among these, LogReg, TabM, XGB, AdaBoost, ExtraTrees, GradBoost, RF, GNB and LDA consistently maintained mean AUC values above 0.85 across the 54 feature-selection combinations (Fig. 2c). Detailed performance metrics for all 756 model configurations are reported in Supplementary Table 2.

Given the primary objective of early disease screening, sensitivity (recall) was prioritized as the key performance metric. Among all evaluated classifiers, LogReg exhibited the highest sensitivity, achieving a mean recall of 0.73 ± 0.07 in identifying individuals who subsequently developed gout. Accordingly, we selected the LogReg model combining median–robust preprocessing, *F*-test based initial screening, and RFE based refined feature selection (mean ROC-AUC > 0.85 with the highest recall) as the optimal screening configuration for gout. Despite the availability of more complex nonlinear models, logistic regression consistently achieved the best balance between sensitivity, discrimination, and stability across feature sets, supporting its suitability for early disease screening. Next, this model configuration was further refined through forward feature selection, Bayesian hyperparameter optimization, and subsequent model interpretability analyses.

### Refinement and interpretation of the proteomic risk signature

**Fig. 3:**
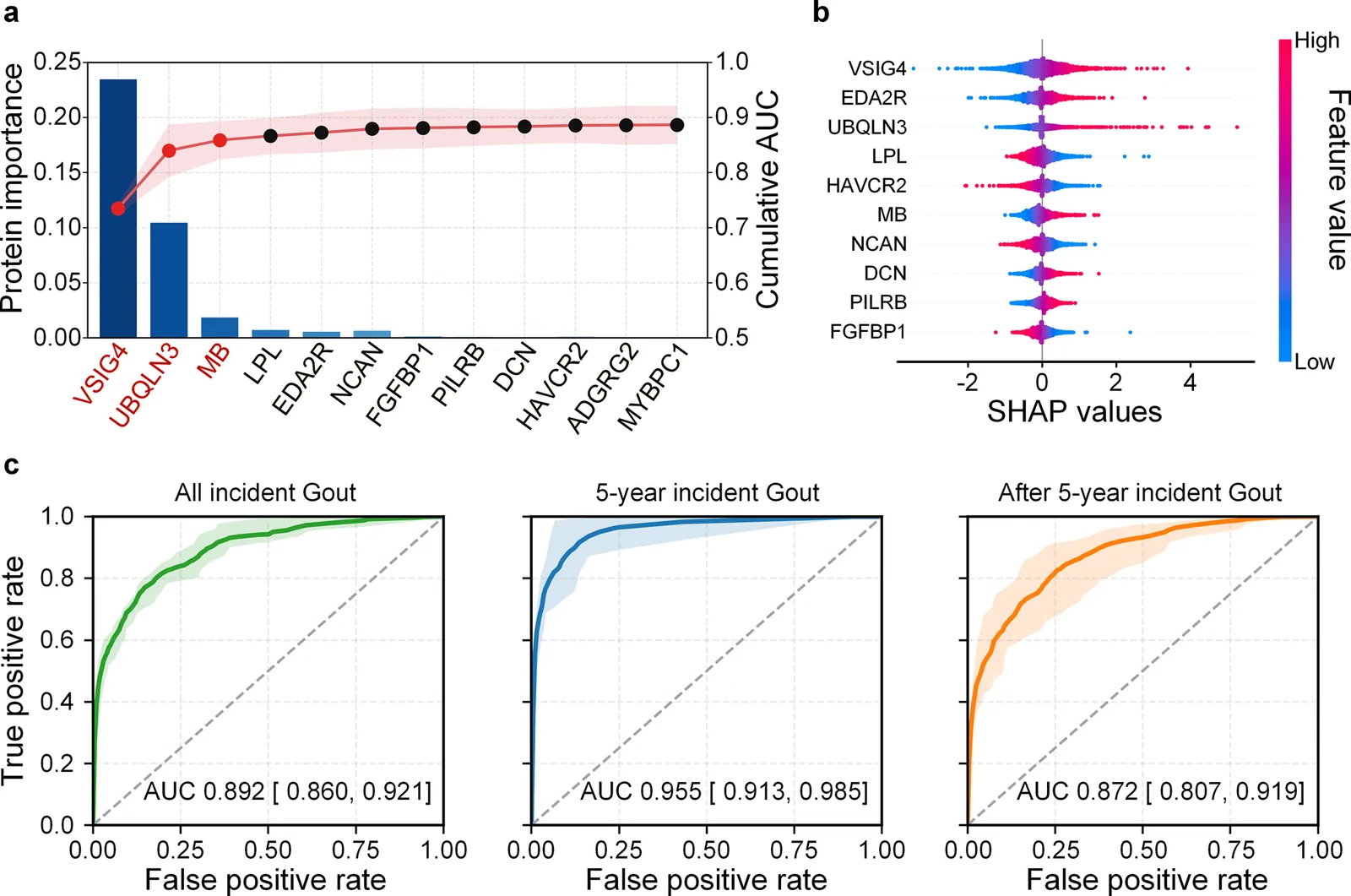
Feature selection and interpretation of the proteomic risk model. **a**, Forward feature selection curve showing incremental changes in cross-validated ROC-AUC as proteins were sequentially added to the model, ordered by importance. **b**, Shapley additive explanation (SHAP) summary plot illustrating the contribution of individual proteins to model predictions. Each point represents an individual participant; color indicates relative protein expression (blue, low; red, high), and horizontal position reflects the direction and magnitude of effect on predicted gout risk. **c**, Receiver operating characteristic (ROC) curves for prediction of overall incident gout, short-term onset (≤5 years), and long-term onset (>5 years), with shaded areas representing 95% confidence intervals

Using the optimal LogReg configuration, we performed forward feature selection to derive a parsimonious signature (Fig. 3a). The model achieved an ROC-AUC > 0.85 with just three proteins (VSIG4, UBQLN3 and MB). Performance plateaued after the inclusion of ten proteins, yielding a final cross-validated ROC-AUC of 0.892 (95% CI: 0.860–0.921). This ten-protein signature provided comparable discrimination to larger feature sets, ensuring model efficiency.

SHAP analysis elucidated the directionality and magnitude of specific protein contributions (Fig. 3b). Global SHAP rankings showed that a limited number of proteins accounted for most of the predictive signal, with higher or lower expression levels consistently associated with increased or decreased predicted gout risk, respectively. Protein importance rankings were consistent across cross-validation folds. We further stratified performance by time to diagnosis. The proteomic model exhibited superior predictive accuracy for short-term onset (≤5 years; ROC-AUC = 0.955) compared to long-term onset (>5 years; ROC-AUC = 0.872) (Fig. 3c). These data suggest that the identified proteomic signature captures an escalating molecular perturbation that intensifies as clinical onset approaches.

### Risk stratification and prognostic value

**Fig. 4:**
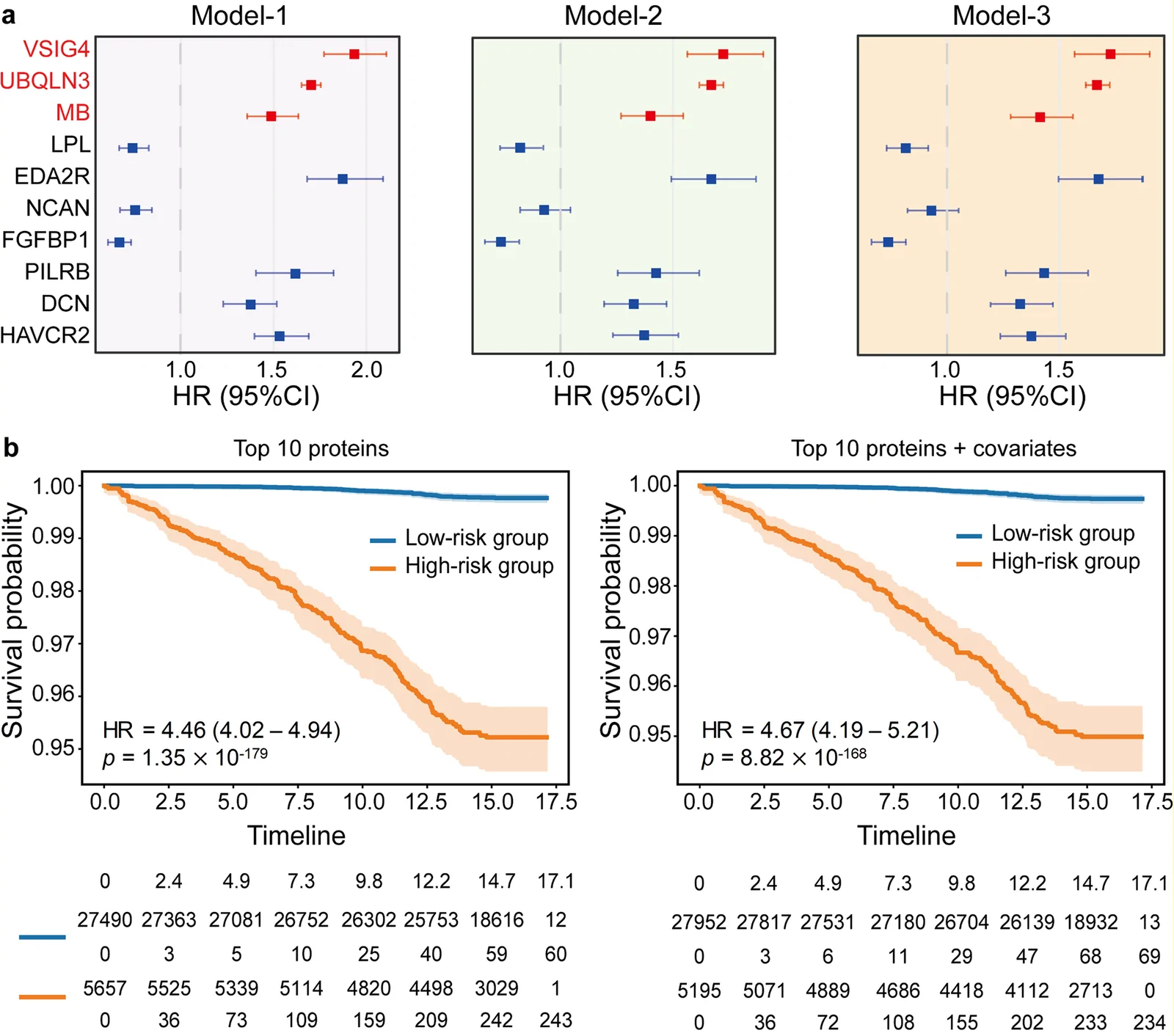
Risk stratification based on the proteomic risk score. **a**, Forest plots showing hazard ratios (HRs) and 95% confidence intervals for the top-ranked proteins derived from Cox proportional hazards models with progressive covariate adjustment. **b**, Kaplan–Meier curves comparing gout-free survival between high- and low-risk groups defined by the proteomic risk score in protein-only and protein-plus-covariate models. Shaded areas denote 95% confidence intervals, and tables indicate numbers at risk over time

To assess clinical utility, we constructed Cox proportional hazards models using the ten-protein signature. All ten proteins were significantly associated with incident gout (*p* < 0.001), with VSIG4, UBQLN3, and MB all exhibiting strong positive associations (Fig. 4a).

These associations remained stable after sequential adjustment for demographic and clinical covariates, indicating that the proteomic signal is independent of traditional risk factors. In contrast, several proteins, including LPL, NCAN, and FGFBP1, exhibited consistent protective associations across models, highlighting heterogeneity in the biological pathways contributing to gout development. Detailed effect estimates for all proteins across the three Cox models are provided in Supplementary Table 3.

Using the proteomic risk scores, participants were stratified into high-risk and low-risk groups according to the predefined cutoff (0.45 for protein-only model, 0.49 for adjustment for conventional covariates). Kaplan–Meier analysis revealed early and sustained divergence in gout-free survival (Fig. 4b). In the protein-only model, individuals in the high-risk group exhibited a markedly elevated hazard of developing gout compared with the low-risk group (HR = 4.46, 95% CI = [4.02–4.94]; *p* = 1.35 × 10^− 179^). This magnitude reflects strong risk stratification rather than causal effect sizes of individual proteins. This strong risk discrimination persisted after adjustment for conventional covariates, with a comparable effect size observed in the protein-plus-covariate model (HR = 4.67, 95% CI = [4.19–5.21]; *p* = 8.82 × 10^− 168^).

The numbers at risk and cumulative event counts further supported the robustness of this stratification (Fig. 4b, bottom tables). Although the high-risk group represented a minority of participants (17.0% in the protein-only model and 15.7% in the protein-plus-covariate model), it consistently exhibited a higher incidence of gout throughout the follow-up period, whereas the low-risk group maintained a consistently low event rate. Collectively, these findings highlight the signature’s capacity to identify a subset of normouricaemic individuals with an imminent high risk of disease progression.

### Temporal trends of predictors in the models

**Fig. 5:**
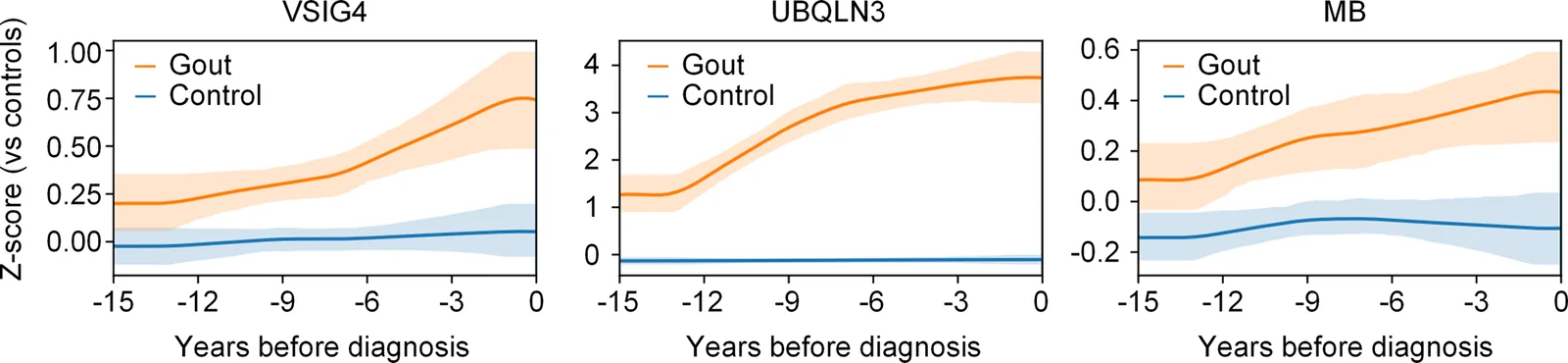
Longitudinal trajectories of key proteins preceding gout diagnosis. Temporal trends of selected proteins over the 15 years before gout diagnosis in a nested case–control analysis. Protein levels were standardized as z-scores relative to matched controls within each time window. Solid lines represent locally estimated scatterplot smoothing (LOESS) curves, and shaded regions denote 95% confidence intervals

In the nested case–control analysis, we profiled the prediagnostic trajectories of the three strongest proteomic predictors (VSIG4, UBQLN3, and MB) across the 15 years preceding diagnosis (Fig. 5). Protein levels were standardized to z-scores against matched controls within each time window, and smoothed trends were estimated using locally weighted regression. All three proteins showed sustained separation between future gout cases and controls long before diagnosis, but with distinct temporal patterns. VSIG4 levels diverged more than a decade prior to diagnosis, showing a progressive increase. UBQLN3 displayed the most pronounced and consistently elevated trajectory, with case–control separation that widened over time and remained high throughout the preclinical period. MB showed a modest but persistent elevation that became more pronounced closer to diagnosis. Notably, none of the proteins exhibited an abrupt spike immediately before diagnosis; rather, they followed smooth, long-term trends, indicating that proteomic signature reflects a chronic, cumulative preclinical pathology rather than an acute reaction to imminent crystallization, supporting the existence of a prolonged “pre-gout” risk state in normouricaemic individuals. Trajectory plots for all ten proteins are presented in Supplementary Fig. 1.

### Specificity analyses using osteoarthritis as a negative control outcome

To assess whether the identified proteomic signature reflects a gout-specific preclinical risk state rather than nonspecific confounding, we conducted a negative control outcome analysis using incident osteoarthritis (OA). Consistent with the primary gout analysis, we restricted this evaluation to participants with normal baseline serum urate. We excluded individuals with prevalent OA or gout at baseline, as well as those who developed gout during follow-up, to minimize overlap with gout-related pathways. The resulting cohort included 32,428 participants, among whom 3,230 developed incident OA.

**Fig. 6:**
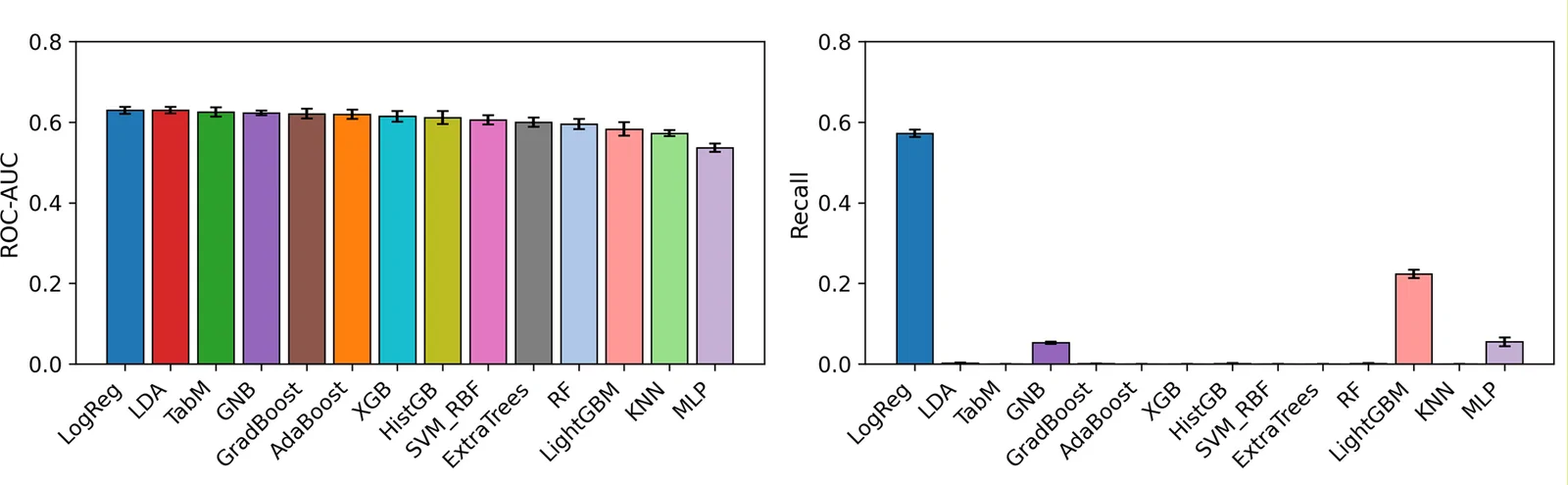
OA negative control outcome analysis. Cross-validated ROC-AUC (left) and recall (right) across 14 models using the same ten-protein signature and median–robust preprocessing as in the primary gout analysis. Discrimination for OA was modest (ROC-AUC ~0.60; best 0.63), supporting outcome specificity

We applied the same 10-protein signature, identical preprocessing pipeline, and the full panel of 14 machine-learning models used in the gout prediction framework. In contrast to the strong discrimination observed for incident gout (ROC-AUC ≈ 0.89), predictive performance for OA was consistently modest across all algorithms, with mean ROC-AUC values clustering around ~0.60 (best model ROC-AUC = 0.63; Fig. 6).

Notably, while discrimination was slightly above chance, classification performance for OA was generally weak, with most models showing very low recall and F1-scores under default probability thresholds, reflecting the substantial class imbalance in this negative control setting. Together, these results support the outcome specificity of the proteomic signature, indicating that it does not generalize as a broadly nonspecific predictor of unrelated musculoskeletal disease, but instead carries more specific risk information for incident gout beyond serum urate.

## Discussion

In this study, we demonstrate that a distinct plasma proteomic signature captures a prolonged molecular pre-gout state, detectable up to 15 years before clinical diagnosis and independent of baseline serum urate levels. By focusing on an initially normouricaemic population of 33,147 individuals and employing a robustness-first machine-learning framework, we identified a parsimonious ten-protein panel that predicts incident gout with high consistency. These findings are supported by multiple complementary layers of validation, including feature stability across 756 model configurations, time-to-event analyses, and long-term prediagnostic trajectories. Collectively, our data support a conceptual shift in understanding gout susceptibility: beyond a disease solely defined by urate saturation, gout risk reflects upstream systemic perturbations in immune regulation and proteostasis that precede overt hyperuricaemia and crystal-driven inflammation.

### A prolonged preclinical susceptibility state beyond serum urate

A central insight of this work is the identification of stable proteomic alterations that predate gout onset by a decade or more, even among individuals with normal serum urate at baseline. This temporal dissociation challenges the traditional paradigm that gout risk emerges only after sustained hyperuricaemia. Instead, it aligns with accumulating evidence across chronic diseases, including rheumatoid arthritis, cardiovascular disease, and metabolic cancers, where prolonged molecular prodromes precede clinical phenotypes [4, 11, 15–18, 20, 35]. Our results situate gout within this broader category of conditions characterized by early systemic reprogramming rather than abrupt biochemical transitions. Crucially, the observed proteomic trajectories represent a gradual and persistent divergence between future cases and controls, suggesting that these markers reflect enduring biological states, such as altered immune tone or metabolic stress, that progressively intensify over time [17, 33, 34, 36].

While serum urate is the obligatory substrate for MSU formation, it remains an insufficient and temporally imprecise predictor of disease onset [7, 8]. By design, our study isolates risk signals independent of pre-existing hyperuricaemia, allowing interrogation of mechanisms that may prime individuals for future disease. The proteins prioritized by our models do not map exclusively to purine metabolism, but instead implicate pathways related to innate immune regulation, cellular clearance, and metabolic stress [4, 8]. This is consistent with the view of gout as a complex autoinflammatory disorder in which the disease threshold is determined not only by crystal burden, but also by the host’s distinct immunometabolic state [11, 37–39]. Such a “primed” susceptibility milieu may exist long before serum urate levels rise to saturation, potentially explaining why some individuals develop gout while others with similar urate burdens remain asymptomatic. This interpretation is further supported by recent UK Biobank metabolomics showing that glycine and glutamine are inversely associated with future gout risk independent of serum urate, with Mendelian randomization evidence implicating upstream metabolic–immune mechanisms rather than urate alone [40].

### Biological context of key predictive proteins

Although our machine-learning approach is primarily data-driven, the biological functions of the top-ranking proteins, VSIG4, UBQLN3, and MB, resonate with established mechanisms of gout pathogenesis, offering hypotheses for future mechanistic validation. While our analyses are predictive rather than mechanistic, a brief biological contextualization of these proteins aligns them with known gout biology.

VSIG4 is exclusively expressed on tissue-resident macrophages and plays a critical role in clearing C3b-opsonized pathogens and dampening T-cell activation [41, 42]. Given that MSU crystals activate the complement system and that macrophages are the primary effectors of the NLRP3 inflammasome in gout [8, 43], elevated VSIG4 may reflect a compensatory upregulation of clearance mechanisms in response to early, subclinical immune irritation or an altered macrophage activation state predisposing to hyper-responsiveness.

UBQLN3, as a member of the ubiquilin family, links polyubiquitinated proteins to the proteasome for degradation. The ubiquitin-proteasome system is pivotal in regulating NLRP3 inflammasome activation and degrading inflammatory mediators [44]. Persistent elevation of UBQLN3 could signal chronic stress on cellular proteostasis (protein quality control), a condition known to lower the threshold for autoinflammatory responses [45].

Beyond its classical role as a marker of muscle injury, elevated circulating MB in subclinical chronic disease may implicate generalized oxidative stress or subtle metabolic dysfunction within skeletal muscle, a key site of insulin resistance [46]. This aligns with the well-known link between metabolic syndrome and gout [47], suggesting that metabolic tissue stress may predate the hyperuricaemic phenotype. Importantly, we interpret these proteins not necessarily as solitary causal drivers, but as integrated sentinels of a systemic “susceptibility milieu” involving innate immune dysregulation and metabolic stress.

### Robustness, clinical stratification, and outcome specificity

A recurrent challenge in high-dimensional omics-based prediction is distinguishing reproducible biological signal from model-specific artifacts [48]. To address this, we adopted a robustness-first analytic strategy, systematically evaluating hundreds of model configurations across preprocessing schemes, feature selection strategies, and classifiers [29]. Rather than optimizing a single algorithm, we emphasized consistency of feature importance and predictive performance across models, an approach increasingly recognized as essential for reliable inference in high-dimensional settings [29, 48]. The emergence of a small, stable set of dominant proteins across diverse analytic choices, together with clear performance plateaus after inclusion of approximately ten features, argues against overfitting and supports the presence of a genuine underlying signal. Notably, simpler models such as logistic regression achieved performance comparable to or exceeding that of more complex algorithms, highlighting that interpretability and generalizability need not be sacrificed for discrimination [49].

From a translational perspective, this signature enabled identification of a high-risk subgroup of normouricaemic individuals (HR > 4) who would be missed by current screening guidelines. While these hazard ratios reflect risk stratification rather than causal effect sizes, they highlight the potential of plasma proteomics to complement serum urate monitoring and expand the window for precision prevention.

Importantly, we further supported outcome specificity through a negative control analysis using incident osteoarthritis. When the same ten-protein signature and modelling framework were applied to OA, predictive discrimination was substantially reduced (ROC-AUC ~0.60; best model ROC-AUC 0.63), compared with the strong performance observed for gout. This attenuation reduces concern that the model is driven by a broadly nonspecific proteomic risk signal, supporting greater outcome specificity for gout susceptibility beyond serum urate.

## Limitations and future directions

Several limitations should be acknowledged. First, gout ascertainment in UK Biobank is primarily based on coded diagnoses rather than systematic outpatient chart review, which may introduce some degree of misclassification. Second, while the UK Biobank provides a massive sample size, external validation in diverse ancestry groups remains essential to ensure generalizability. Third, proteomic measurements were baseline-only; longitudinal sampling would provide finer resolution of temporal dynamics. Finally, causal inference was beyond the scope of this work. Integration with genetic instruments, functional studies, and experimental models will be necessary to disentangle cause from consequence and to assess whether any identified proteins lie on modifiable causal pathways.

Future work should also explore multi-omics integration, combining proteomics with metabolomics, polygenic risk scores, and longitudinal clinical data to refine risk prediction and biological interpretation. Such efforts could help distinguish molecular subtypes of gout susceptibility and inform tailored prevention strategies.

## Conclusions

In summary, large-scale plasma proteomics in 33,147 normouricaemic individuals identifies a stable molecular pre-gout state detectable more than a decade before clinical onset. A parsimonious 10-protein signature enables prospective risk stratification beyond the traditional serum urate paradigm. These findings provide a framework for precision prevention and earlier intervention in individuals currently considered low-risk by conventional biochemical standards.

## Electronic supplementary material

Below is the link to the electronic supplementary material.

Supplementary Material 1

Supplementary Material 2
