# Supplementary material for: Identification of a 10-protein plasma signature for preclinical gout risk beyond serum urate: a robustness-first proteomic study in 33,147 normouricaemic individuals
Source: J Transl Med. 2026 Jun 24;24:1239. doi: 10.1186/s12967-026-08505-3 (PMC13637303; doi:10.1186/s12967-026-08505-3)
Supplement: Supplementary file 1 [file 12967_2026_8505_MOESM1_ESM.docx]

**Supplementary Fig. 1 Associations between the selected 10 proteins and incident gout in competing-risk analyses.**

Fine–Gray subdistribution hazard models were fitted with all-cause death before gout onset treated as a competing event. Subdistribution hazard ratios are shown with 95% confidence intervals. Models 1–3 used the same covariate adjustments as the corresponding Cox models.

**Supplementary Fig. 2 PPV and NPV curves of the prediction models.**

Positive predictive value, negative predictive value, sensitivity, and specificity are shown across probability thresholds. Dashed vertical lines indicate the Youden-derived thresholds used for risk stratification: 0.45 for the protein-only model and 0.49 for the protein-plus-covariate model. A, protein-only model; B, protein-plus-covariate model.

**Supplementary Fig. 3 Longitudinal trajectories of top 10 proteins preceding gout diagnosis.**

Temporal trends of selected proteins over the 15 years before gout diagnosis in a nested case–control analysis. Protein levels were standardized as z-scores relative to matched controls within each time window. Solid lines represent locally estimated scatterplot smoothing (LOESS) curves, and shaded regions denote 95% confidence intervals.

**Supplementary Fig. 4. PPI network analysis of the identified 10-protein signature using the STRING database.**

STRING analysis showed modest overall connectivity, with partial clustering involving VSIG4–HAVCR2 and DCN–NCAN–LPL. These subnetworks were broadly related to immune regulation, extracellular matrix organization, and lipid-associated processes, whereas several proteins remained relatively isolated. This pattern suggests that the signature captures multiple complementary biological dimensions of preclinical gout susceptibility rather than a single tightly connected pathway.
